# NF-kB (p50/p65)-Mediated Pro-Inflammatory microRNA (miRNA) Signaling in Alzheimer's Disease (AD)

**DOI:** 10.3389/fnmol.2022.943492

**Published:** 2022-06-28

**Authors:** Walter J. Lukiw

**Affiliations:** ^1^LSU Neuroscience Center, Louisiana State University Health Science Center, New Orleans, LA, United States; ^2^Department of Ophthalmology, Louisiana State University Health Science Center, New Orleans, LA, United States; ^3^Department Neurology, Louisiana State University Health Science Center, New Orleans, LA, United States

**Keywords:** Alzheimer's disease (AD), microRNA, NF-kB (p50/p65), reactive oxygen species (ROS), lipopolysaccharide, miRNA-based therapy, miRNA-9/miRNA-30b/miRNA-34a/miRNA-146a/miRNA-155, anti-NF-kB therapy

## Introduction

A small family of NF-kB (p50/p65)-regulated microRNAs (miRNAs) has been identified and partially characterized in Alzheimer's disease (AD) brain in the hippocampal CA1 and temporal lobe neocortical regions, and in reactive oxygen species (ROS)-, cytokine interleukin 1-beta (IL-1β)-, amyloid beta 42 (Aβ42) peptide- and/or lipopolysaccharide (LPS)-stressed human neuronal-glial (HNG) cells in primary co-culture. This microRNA family currently includes miRNA-9, miRNA-30b, miRNA-34a, miRNA-125b, miRNA-146a and miRNA-155. Other brain-enriched miRNA species may be involved. Experimental evidence suggests that in neurological diseases, the upregulation of this small pro-inflammatory miRNA family orchestrates a pathogenic gene expression program that can explain many aspects of AD onset, and propagation and severity of the disease including failure of microglial-mediated clearance of amyloid-beta (Aβ) and other end-stage peptides from brain cells, amyloidogenesis, astrogliosis, deficits in neurotropism, neuronal cell atrophy, and downregulation in the production of essential cytoskeletal components and synaptic signaling elements. This opinion article will contribute to the interpretation of the most recent findings in the current research area involving NF-kB-regulated miRNA-mediated signaling pathways, and how this information may aid in the advancement of therapeutic strategies for more effective clinical management of AD and other progressive age-related and lethal neurodegenerative disorders.

## Alzheimer's Disease (AD): Epidemiology and Basic Features

AD is a slowly developing, irreversible, progressive, and inflammatory neurodegeneration of the human brain, central nervous system (CNS), and neural vasculature (Alzheimer et al., 1995; Lane et al., 2018; Trejo-Lopez et al., 2021). Due in part to the aging population and demographics, the global incidence and prevalence of AD are sharply increasing, and AD currently represents the largest single cause of age-related memory impairment and behavioral and cognitive decline in Westernized societies. Current estimates put the global incidence of AD at about ~55 million, and this number is expected to reach epidemic proportions and rise to about ~150 million cases by the year 2050 (Tahami Monfared et al., 2022; https://alz-journals.onlinelibrary.wiley.com/doi/full/10.1002/alz.12638; https://www.alz.org/media/documents/Alzheimers-facts-and-figures.pdf; https://www.Alzint.org/about/dementia-facts-figures/dementia-statistics/last accessed 13 June 2022). AD places a tremendous socioeconomic burden on caregivers and on the healthcare system as a whole. Despite ~115 years of intense and directed research, no effective treatment or cure for AD exists, although many insights into the molecular-genetic nature of this age-related disease have been established (Alzheimer et al., 1995; Kaur et al., 2015; Lane et al., 2018; Dong et al., 2021; Hermans et al., 2021; Trejo-Lopez et al., 2021; Liu et al., 2022; Olufunmilayo and Holsinger, 2022; Pogue et al., 2022; Rastegar-Moghaddam et al., 2022; Singh et al., 2022; Tahami Monfared et al., 2022; Yoon et al., 2022). As a devastating, inflammatory, and terminal neurodegenerative disorder, one molecular genetic component that has emerged at the forefront of current AD research is the DNA-binding element NF-kB, probably the single most important transcription factor yet identified in the complex neuropathology of AD. This opinion article will address some recently described effects of NF-kB on a small family of regulatory miRNAs observed to be significantly altered in abundance, complexity and speciation in AD-affected brain.

## NF-κB-Sensitive microRNAs and Inflammatory Signaling in Alzheimer's Disease (AD)

Discovered about ~36 years ago, the heterodimeric transcription factor NF-kB (p50/p65) was first described as a rapidly inducible nuclear factor κ-light-chain transcription enhancer of activated B cell lymphocytes in part responsible for the humoral immunity component of the immune system (Sen and Baltimore, 1986; Kaltschmidt et al., 1993; Taganov et al., 2006). Since then, recognition sites for this redox-controlled transcriptional activator and DNA-binding protein have been found to occur in multiple gene promoters, and it is now known to drive a broad range of gene expression patterns in diverse cellular types involved in host innate- and adaptive-immune functions with important roles in the clearance of waste molecules from the cytoplasm, inflammatory signaling, differentiation, cell growth, tumorigenesis and neurodegeneration (Taganov et al., 2006; Zhang et al., 2017; Baltimore, 2019). In resting cells the pre-formed NF-kB (p50/p65; 115 kDa) heterodimer is bound to a hydrophobic inhibitory kappa B protein (IkB, 36 kDa) that prevents free NF-kB from promoter-DNA-binding and gene transactivation. However NF-κB can be rapidly induced in all cell types by treating them with pro-inflammatory mediators that include interleukin-1beta (IL-1β), interleukin 6 (IL-6), tumor necrosis factor alpha (TNFα), and endotoxins such as lipopolysaccharides (LPS), an intensely inflammatory lipoprotein produced exclusively by Gram-negative bacteria, Aβ peptides, viral gene products, ultraviolet and other ionizing irradiation, and reactive oxygen species (ROS)-generating and/or oxidizing compounds. Exposure to these factors induces IkB decomposition triggered through a site-specific phosphorylation of an IκB kinase (IKK) complex, followed by degradation via the ubiquitin-proteasome system, and then rapid NF-kB-activated transcription by multiple pro-inflammatory NF-kB-sensitive gene promoters. The human brain and CNS contain a very active, pleiotropic NF-kB-signaling system that has a function in both neurological health and disease (Kaltschmidt and Kaltschmidt, 2009; Christian et al., 2016; Liu et al., 2022; Yoon et al., 2022). While the NF-kB (p50/p65; also known as p50/RelA) heterodimer is especially abundant in nervous tissues, other homo- or hetero-dimeric NF-kB transcription factor complexes containing at least 5 other molecular components that include RelA, RelB, c-Rel, p50, p52, and NFKB1 (p105) form at least 12 different identified NF-kB complexes. All share the conserved Rel homology domain (RHD) responsible for DNA binding, dimerization, and association with the repressor protein IκB (Zhang et al., 2017; Pires et al., 2018; Baltimore, 2019; https://www.genecards.org/cgi-bin/carddisp.pl?gene=RELA; last accessed 25 May 2022). It has been repeatedly demonstrated: (i) that activated NF-kB subsequently upregulates a select sub-group of disease-associated miRNAs comprising a well-characterized NF-kB-sensitive *Homo sapiens-*enriched family of potentially pathogenic miRNAs that include miRNA-9, miRNA-30b, miRNA-34a, miRNA-146a, and miRNA-155 normally involved in immunity, inflammation, and brain cell genetic function in the CNS (Lukiw, 2012, 2020; Brennan et al., 2019; Juźwik et al., 2019; Barnabei et al., 2021; Song et al., 2021; Yoon et al., 2022); (ii) that the same miRNAs are upregulated in AD brain; (iii) that this group of significantly over-expressed miRNAs are abundant in progressive and often lethal viral and prion-mediated and/or related neurological syndromes associated with progressive inflammatory neurodegeneration; and (iv) that all of these NF-kB-sensitive endotoxin-responsive miRNA genes have as many as three tandem, functional NF-kB binding sites stacked in their immediate 5'-promoter region, making this miRNA gene family exceptionally sensitive to NF-kB activation and NF-kB-mediated transcriptional upregulation (Taganov et al., 2006; Lukiw et al., 2008; Alexandrov et al., 2019; Jauhari et al., 2020; Table 1). Interestingly, there is also abundant recent evidence (i) that endotoxins such as LPS and other biophysical-barrier-penetrating glycolipids and lipoproteins specifically induce neurodegeneration by promoting neuro-inflammatory signaling by stimulation of Toll-like receptors (TLRs) present on the outer membranes of glial cells of the CNS (Kumar, 2019; Singh et al., 2022); (ii) that extremely potent pro-inflammatory species of LPSs have been found by many independent groups to be localized in human brain neurons in AD brain (Zhan et al., 2018, 2021; Alexandrov et al., 2019; Zhao et al., 2019a; Singh et al., 2022); and (iii) that a life-long supply of microbiome-abundant Gram-negative bacteria-derived LPS would be available over the long-term to chronically upregulate NF-kB in the brain and CNS, as is observed in LPS-treated human neurons in primary culture and in AD temporal lobe neocortex, the latter an anatomical area targeted by the AD process (Lukiw and Bazan, 1998; Zhang et al., 2017; Zhan et al., 2018, 2021; Zhao and Lukiw, 2018; Alexandrov et al., 2019; Zhao et al., 2019a; Singh et al., 2022).

**Table 1:** NF-kB-induced microRNAs (miRNAs), their mRNA targets, functions, and pathways in Alzheimer's disease (AD)-affected brain; all miRNAs listed show: (i) significant upregulation following NF-kB (p50/p65) activation; (ii) are potentially pathogenic and are upregulated in AD brain, AD cell culture, and transgenic murine models of AD (TgAD); and (iii) have been shown to interactively contribute to the AD process; some closely related work on the functions these miRNAs in neuronal cell culture, TgAD models, and other human neurodegenerative diseases have also been included in the References.

**Human miRNA**	**mRNA target(s)**	**mRNA function**	**Result of mRNA and gene expression deficit**	**Reference**
miRNA-9	CFH	complement factor H; repressor of activation of the innate immune response in brain and retina at the C3 to C3b transition	CFH deficits in disease are pro-inflammatory; excessive activation of the innate-immune response, altered immune-signaling, neuroinflammation	Clement et al. (2016); Recabarren and Alarcón (2017); Chen et al. (2018); Natoli and Fernando (2018)
	TREM2	triggering receptor expressed in myeloid and microglial cells; surface receptor essential in removing cellular debris; viral infection	deficits in phagocytosis and clearance of end-stage metabolic products from cells including amyloid beta (Aβ) peptides	Lukiw et al. (2008); Zhao and Lukiw (2013, 2018); Bhattacharjee et al. (2016); Chen et al. (2021)
	TSPAN-7	transmembrane spanning superfamily member 7; regulator of surface receptor signal transduction; activates ADAM10-dependent cleavage activity of βAPP; tau seeding activity; viral infection	amyloidogenesis; impairment of autophagic activity and promotion of amyloid plaque formation; inhibition of autophagy; supports the formation of neurofibrillary tangles (NFT)	Jaber et al. (2019); Perot and Ménager (2020); Chen et al. (2021); Turk et al. (2021)
miRNA-30b	NF-L (NEFL)	neurofilament light chain protein; neuron-specific; supports neuronal cytoarchitecture, radial diameter of axons and synaptic signaling	loss of neuronal cytoarchitecture and signaling capacity; neuronal atrophy; synaptic structural deficits; general biomarker for neurodegeneration	McLachlan et al. (1990); Kittur et al. (1994); Brennan et al. (2019); Xu et al. (2021); Zhao et al. (2021)
miRNA-34a	SHANK3	SH3 proline-rich and multiple-ankyrin-repeat domain (SHANK3) cytoskeletal anchoring protein; gene directional gene expression effects; associated with viral infection	Disruption of the cytoskeleton and post-synaptic structure and scaffolding network; deficits in synaptic organization, dendritic spine maturation and synaptic vesicle release	Lu et al. (2010); Choi et al. (2015); Jaber et al. (2017); Lee et al. (2017); Jin et al. (2018); Zhao et al. (2019b)
	TREM2	triggering receptor expressed in myeloid and microglial cells; surface receptor essential in removing cellular debris; associated with viral (SARS-CoV-2) infection	deficits in phagocytosis and clearance of end-stage metabolic products from cells including amyloid beta (Aβ) peptides- deficits in AD and AMD	Zhao and Lukiw (2013); Zhao et al. (2013); Hermans et al. (2021); Wu et al. (2021); Olufunmilayo and Holsinger (2022)
miRNA-125b	CDKN2A	cyclin-dependent kinase inhibitor 2A cell cycle inhibitor; induces cell cycle arrest; viral (SARS-CoV-2) infection	downregulation of cell cycle control: astrogliosis and glial cell proliferation	Pogue et al. (2010); Czapski et al. (2021); Valeri et al. (2021)
	SYN-2	synapsin-2: neuron-specific; neuronal synaptic phosphoprotein; coats synaptic vesicles; functions in the regulation of neurotransmitter release	impairment of neurotransmitter release and trans-synaptic signaling; synaptic signaling deficits observed in AD brain and in TgAD murine models	Ferreira and Rapoport (2002); Mirza and Zahid (2018); Zhao et al. (2019a, 2021); Jeśko et al. (2021)
	15-LOX-1	ALOX15; arachidonate 15-lipoxygenase; essential in the conversion of docosahexaenoic acid to neuro-protectin D1 (NPD1)	lack of NPD1; lack of neurotrophic support; deficiency of neurotrophic omega-3 fatty acid derivatives in the human brain neocortex	Lukiw et al. (2005); Zhao et al. (2014); Sun et al. (2018); Al-Fadly et al. (2019)
miRNA-146a	CFH	complement factor H; complement control glycoprotein; deficits in disease are pro-inflammatory to the host; viral (SARS-CoV-2) infection; see above	defective control and stimulation of the innate- immune response; and pro-inflammatory signaling; role in neuro-pathological signaling in AD and prion disease	Alexandrov et al. (2019); Slota and Booth (2019); Fan et al. (2020); Yu et al. (2020); Aslani et al. (2021); Pogue and Lukiw (2021); Wu et al. (2021)
	IRAK-1	interleukin-1 receptor-associated kinase 1; initiation and sustenance of the innate immune response and NF-κB signaling	compensatory surge in IRAK-2 and chronic stimulation of NF-κB signaling in the brain; neuroinflammation	Cui et al. (2010); Arena et al. (2017); Aslani et al. (2021)
	TSPAN12	transmembrane superfamily member 12; cell surface receptor involved in signal transduction; activates ADAM10-dependent cleavage of βAPP and tau seeding activity	pathological shift from neurotrophic (sAPPα) to amyloidogenic (beta- and gamma-secretase cleavage); processing of βAPP into neurotoxic Aβ42 peptides	Li et al. (2011); Jaber et al. (2017); Aslani et al. (2021); Miyoshi et al. (2021)
miRNA-155	CFH	complement factor H; see above	defective regulation of the innate-immune response (see above)	Chen et al. (2018); Zingale et al. (2021); Rastegar-Moghaddam et al. (2022)

## Therapeutic Strategies Directed Against NF-kB and/or Pro-Inflammatory miRNA Signaling

Because NF-kB signaling pathways appear to be centrally involved in a great many immune- and inflammation-linked human diseases from carcinogenesis to neurodegeneration, a large number of natural and synthetic NF-kB inhibitors have been designed and tested, and are under very active investigation to limit the extent of NF-kB activation in the cellular environment. The evolution of one single high-potency gene-activating transcription factor in overlapping signaling pathways has made it challenging to find biologically active inhibitory molecules that can interfere with and/or block specific signaling pathways that lead to NF-κB activation without multiple off-target effects or other complicating factors. Even so, a number of NF-kB modulator and blocking strategies have been adopted or are undergoing rigorous evaluation in both research laboratories and in clinical settings. In general, therapeutic NF-kB inhibition can occur *via* four basic mechanistic strategies: (i) by blockage of the original physiological stimulating signals that drive NF-kB activation; (ii) by targeting phosphorylation pathways associated with NF-kB activation because NF-κB phosphorylation controls transcription in a gene-specific manner; (iii) by modulation or activation of the IkB complex or other NF-kB subunits; and/or (iv) by blockage of NF-kB translocation, DNA sequence recognition, and binding and/or modification of NF-kB that affects its activity or target specificity. These approaches include but are not limited to the following strategies, most of which are under very active research and therapeutic development: (i) antioxidant approaches that neutralize the primary NF-kB activation signals such as quenching of reactive oxygen species (ROS) and other oxidizing and/or redox compounds (Kaur et al., 2015; Barnabei et al., 2021; Jover-Mengual et al., 2021; Pogue and Lukiw, 2021); (ii) alteration of multiple phosphorylation events that disrupt the activation of the multi-subunit NF-kB transcription complex and alter the interaction of these phosphorylation sites that ultimately determines the selectivity of NF-κB effects on transcriptional activity (by advanced investigation of these highly interactive phosphorylation sites and mechanisms it should be possible to modulate or block many aspects of phosphorylation-mediated NF-kB activation; Christian et al., 2016; https://360researchreports.com/global-nf-kb-inhibitors-market-20149725); (iii) as previously pointed out, because active NF-kB complexes are assembled from various combinations of RelA, RelB, c-Rel, p50, p52, NFKB1 (p105) etc., it may be possible to specifically block the generation and/or assembly of a single monomeric species of NF-kB by limiting the abundance of one of the subunits of NF-kB by genome editing, including knock-out technologies and/or the Cas9/CRISPR editing system (Wang et al., 2018; Dai et al., 2020; Katti et al., 2022); (iv) NF-kB is directed to bind to its genomic targets by topological features located in certain promoter DNA sequences; therefore, these highly specific promoter DNA binding sites can be masked, blocked, or modulated using genome blocking technologies involving small non-coding RNA (sncRNA), NF-kB decoy sequence, and/or Cas9/CRISPR editing strategies (Taganov et al., 2006; Christian et al., 2016; Zhang et al., 2017; Baltimore, 2019; Dai et al., 2020; Katti et al., 2022; Yoon et al., 2022); (v) specific upregulated miRNA abundance and speciation may be blocked or modulated using chemically stabilized anti-miRNA strategies or by targeting miRNA processing enzymes, thus preventing the creation of a fully active and/or biologically available miRNA species; (vi) directed delivery systems to the brain and/or CNS or other tissue and/or organ systems to minimize unwarranted off-target effects; (vii) long-term systemic ingestion of low-dose NF-κB inhibitors including dietary-administered flavonoids, lignans, diterpenes and sesquiterpenes, saponins, polysaccharides, polyphenols, biological fiber, and other natural products that may have general beneficial effects on reducing pro-inflammatory signaling, carcinogenesis, and neurodegeneration by chronic dampening of NF-κB activities. These have been used for millennia in the pharmacopeia associated with ethnic biomedical-treatment approaches (Gilmore and Herscovitch, 2006; Li et al., 2020a,b; Olajide and Sarker, 2020; Uddin et al., 2021; Al-Khayri et al., 2022; Das et al., 2022); and/or (viii) any combination of these therapeutic strategies. Regarding the creation and testing of synthetic NF-kB modulatory or blocking compounds, about ~80 pharmaceutical companies are currently in the pursuit of more effective, directed and specifically targeted NF-kB inhibitors for clinical application in human diseases involving excessive NF-kB activation and signaling (https://www.futuremarketinsights.com/reports/nf-kb-inhibitors-market; https://www.Globenewswire.com/news-release/2020/12/08/2141 093/0/en/NF-kappa-B-Inhibitors-Therapeutics-pipeline-analysis-of-80-Companies.html; https://360researchreports.com/global-nf-kb-inhibitors-market- 20149725; last accessed 25 May 2022).

## Discussion

The pleiotropic homo- or hetero-dimeric transcription factor NF-kB is a master regulator of the innate immune system, inflammatory responses, and neurotropic, cytoskeletal, synaptic, phagocytic, and synaptic deficit characteristics of the AD-affected brain and related neurodegenerative disorders. One critically important regulatory action of the NF-kB complex is to upregulate a small family of pathogenic miRNAs in the brain and CNS, which in turn targets and downregulates a group of mRNAs whose downregulation is decisive in driving the neuropathology of AD (Table 1). It is our opinion that these NF-kB-regulated miRNA-mRNA linked pathological interactions will provide a wealth of targets for therapeutics and disease intervention. The merging of experimental and human clinical trials will be decisive in the strategic design and application of NF-kB and NF-kB-miRNA-induced modulatory agents and drugs useful in the more efficacious treatment of the current AD epidemic.

## Author Contributions

WL researched, compiled data, and wrote the paper. WL approved and submitted the final version.

## Funding

Research involving small non-coding RNA (sncRNA) and miRNA signaling, including NF-kB (p50/p65)-mediated miRNA signaling, the innate-immune response, amyloidogenesis and neuro-inflammation in AD and prion disease, contributions of the microbiome-derived neurotoxins such as lipopolysaccharide (LPS), reactive oxygen species (ROS), anti-NF-kB and/or anti-miRNA therapeutic strategies was supported through the Louisiana Biotechnology Research Network (LBRN), an unrestricted grant to the LSU Eye Center from Research to Prevent Blindness (RPB), The Brown Foundation, Joe and Dorothy Dorsett Innovation in Science Healthy Aging Award; and National Institutes of Health (NIH) NIA Grants AG18031 and AG038834.

## Conflict of Interest

The author declares that the research was conducted in the absence of any commercial or financial relationships that could be construed as a potential conflict of interest.

## Publisher's Note

All claims expressed in this article are solely those of the authors and do not necessarily represent those of their affiliated organizations, or those of the publisher, the editors and the reviewers. Any product that may be evaluated in this article, or claim that may be made by its manufacturer, is not guaranteed or endorsed by the publisher.

## References

[B1] Alexandrov P. Zhao Y. Li W. Lukiw W. J. (2019). Lipopolysaccharide-stimulated, NF-kB-, miRNA-146a- and miRNA-155-mediated molecular-genetic communication between the human gastrointestinal tract microbiome and the brain. Folia Neuropathol. 57, 211–219. 10.5114/fn.2019.88449 31588707

[B2] Al-Fadly E. D. Elzahhar P. A. Tramarin A. Elkazaz S. Shaltout H. Abu-Serie M. M.. (2019). Tackling neuroinflammation and cholinergic deficit in Alzheimer's disease: multi-target inhibitors of cholinesterases, cyclooxygenase-2 and 15-lipoxygenase. Eur. J. Med. Chem. 167, 161–186. 10.1016/j.ejmech.2019.02.012 30771604

[B3] Al-Khayri J. M. Sahana G. R. Nagella P. Joseph B. V. Alessa F. M. Al-Mssallem M. Q. (2022). Flavonoids as potential anti-inflammatory molecules: a review. Molecules. 27, 2901. 10.3390/molecules27092901 35566252 PMC9100260

[B4] Alzheimer A. Stelzmann R. A. Schnitzlein H. N. Murtagh F. R. (1995). An English translation of Alzheimer's 1907 paper, “Uber eine eigenartige Erkankung der Hirnrinde”. Clin. Anat. 8, 429–431. 10.1002/ca.980080612 8713166

[B5] Arena A. Iyer A. M. Milenkovic I. Kovacs G. G. Ferrer I. Perluigi M.. (2017). Developmental expression and dysregulation of miRNA-146a and miRNA-155 in Down's syndrome and mouse models of down's syndrome and Alzheimer's disease. Curr. Alzheimer's Res. 14, 1305–1317. 10.2174/1567205014666170706112701 28720071

[B6] Aslani M. Mortazavi-Jahromi S. S. Mirshafiey A. (2021). Efficient roles of miRNA-146a in cellular and molecular mechanisms of neuroinflammatory disorders: An effectual review in neuroimmunology. Immunol. Lett. 238, 1–20. 10.1016/j.imlet.2021.07.004 34293378

[B7] Baltimore D. (2019). Sixty years of discovery. Annu. Rev. Immunol. 37, 1–17. 10.1146/annurev-immunol-042718-041210 30379594

[B8] Barnabei L. Laplantine E. Mbongo W. Rieux-Laucat F. Weil R. (2021). NF-κB: at the borders of autoimmunity and inflammation. Front. Immunol. 12, 716469. 10.3389/fimmu.2021.716469 34434197 PMC8381650

[B9] Bhattacharjee S. Zhao Y. Dua P. Rogaev E. I. Lukiw W. J. (2016). microRNA-34a-mediated down-regulation of the microglial-enriched triggering receptor and phagocytosis-sensor TREM2 in age-related macular degeneration. PLoS One. 11, e0150211. 10.1371/journal.pone.0150211 26949937 PMC4780721

[B10] Brennan S. Keon M. Liu B. Su Z. Saksena N. K. (2019). Panoramic visualization of circulating microRNAs across neurodegenerative diseases in humans. Mol. Neurobiol. 56, 7380–7407. 10.1007/s12035-019-1615-1 31037649 PMC6815273

[B11] Chen J. Qi Y. Liu C. F. Lu J. M. Shi J. Shi Y. (2018). microRNA expression data analysis to identify key miRNAs associated with Alzheimer's disease. J. Gene Med. 20, e3014. 10.1002/jgm.3014 29543360

[B12] Chen M. L. Hong C. G. Yue T. Li H. M. Duan R. Hu W. B.. (2021). Inhibition of miR-331-3p and miR-9-5p ameliorates Alzheimer's disease by enhancing autophagy. Theranostics 11, 2395–2409. 10.7150/thno.47408 33500732 PMC7797673

[B13] Choi S. Y. Pang K. Kim J. Y. Ryu J. R. Kang H. Liu Z. (2015). Post-transcriptional regulation of SHANK3 expression by microRNAs related to multiple neuropsychiatric disorders. Mol. Brain. 8, 74. 10.1186/s13041-015-0165-3 26572867 PMC4647645

[B14] Christian F. Smith E. L. Carmody R. J. (2016). The regulation of NF-κB subunits by phosphorylation. Cells 5, 12. 10.3390/cells5010012 26999213 PMC4810097

[B15] Clement C. Hill J. M. Dua P. Culicchia F. Lukiw W. J. (2016). Analysis of RNA from Alzheimer's disease post-mortem brain tissues. Mol. Neurobiol. 53, 1322–1328. 10.1007/s12035-015-9105-6 25631714 PMC5450164

[B16] Cui J. G. Li Y. Y. Zhao Y. Bhattacharjee S. Lukiw W. J. (2010). Differential regulation of interleukin-1 receptor-associated kinase-1 (IRAK-1) and IRAK-2 by microRNA-146a and NF-kB in stressed human astroglial cells and in Alzheimer disease. J. Biol. Chem. 285, 38951–38960. 10.1074/jbc.M110.178848 20937840 PMC2998119

[B17] Czapski G. A. Cieślik M. Białopiotrowicz E. Lukiw W. J. Strosznajder J. B. (2021). Down-regulation of cyclin D2 in amyloid β toxicity, inflammation, and Alzheimer's disease. PLoS ONE 16, e0259740. 10.1371/journal.pone.0259740 34793515 PMC8601534

[B18] Dai W. Wu J. Wang D. Wang J. (2020). Cancer gene therapy by NF-κB-activated cancer cell-specific expression of CRISPR/Cas9 targeting telomeres. Gene Ther. 27, 266–280. 10.1038/s41434-020-0128-x 32034293

[B19] Das R. Mehta D. K. Dhanawat M. (2022). Medicinal plants in cancer treatment: contribution of nuclear factor-kappa B (NF-kB) inhibitors. Mini. Rev. Med. Chem. 10.2174/1389557522666220307170126 35260052

[B20] Dong Z. Gu H. Guo Q. Liang S. Xue J. Yao F.. (2021). Profiling of serum exosome miRNA reveals the potential of a miRNA panel as diagnostic biomarker for Alzheimer's disease. Mol. Neurobiol. 58, 3084–3094. 10.1007/s12035-021-02323-y 33629272

[B21] Fan W. Liang C. Ou M. Zou T. Sun F. Zhou H.. (2020). microRNA-146a Is a wide-reaching neuroinflammatory regulator and potential treatment target in neurological diseases. Front. Mol. Neurosci. 13, 90. 10.3389/fnmol.2020.00090 32581706 PMC7291868

[B22] Ferreira A. Rapoport M. (2002). The synapsins: beyond the regulation of neurotransmitter release. Cell. Mol. Life Sci. 59, 589–595. 10.1007/s00018-002-8451-5 12022468 PMC11337460

[B23] Gilmore T. D. Herscovitch M. (2006). Inhibitors of NF-kappaB signaling: 785 and counting. Oncogene. 25, 6887–6899. 10.1038/sj.onc 17072334

[B24] Hermans S. J. Nero T. L. Morton C. J. Gooi J. H. Crespi G. A. N. Hancock N. C.. (2021). Structural biology of cell surface receptors implicated in Alzheimer's disease. Biophys. Rev. 14, 233–255. 10.1007/s12551-021-00903-9 35340615 PMC8921391

[B25] Jaber V. Zhao Y. Lukiw W. J. (2017). Alterations in micro RNA-messenger RNA (miRNA-mRNA) coupled signaling networks in sporadic Alzheimer's disease (AD) hippocampal CA1. J. Alzheimer's Dis. Parkinsonism. 7, 312. 10.4172/2161-0460.1000312 29051843 PMC5645033

[B26] Jaber V. R. Zhao Y. Sharfman N. M. Li W. Lukiw W. J. (2019). Addressing Alzheimer's disease (AD) neuropathology using anti-microRNA (AM) strategies. Mol. Neurobiol. 56, 8101–8108. 10.1007/s12035-019-1632-0 31183807 PMC6842093

[B27] Jauhari A. Singh T. Mishra S. Shankar J. Yadav S. (2020). Coordinated action of miRNA-146a and parkin gene regulate rotenone-induced neurodegeneration. Toxicol. Sci. 176, 433–445. 10.1093/toxsci/kfaa066 32392329

[B28] Jeśko H. Wieczorek I. Wencel P. L. Gassowska-Dobrowolska M. Lukiw W. J. Strosznajder R. P. (2021). Age-related transcriptional deregulation of genes coding synaptic proteins in Alzheimer's disease murine model: potential neuroprotective effect of fingolimod. Front. Mol. Neurosci. 14, 660104. 10.3389/fnmol.2021.660104 34305524 PMC8299068

[B29] Jin C. Kang H. Ryu J. R. Kim S. Zhang Y. Lee Y.. (2018). Integrative brain transcriptome analysis reveals region-specific and broad molecular changes in SHANK3-overexpressing mice. Front. Mol. Neurosci. 11, 250. 10.3389/fnmol.2018.00250 30233305 PMC6127286

[B30] Jover-Mengual T. Hwang J. Y. Byun H. R. Court-Vazquez B. L. Centeno J. M. Burguete M. C.. (2021). The role of NF-κB triggered inflammation in cerebral ischemia. Front. Cell. Neurosci. 15, 633610. 10.3389/fncel.2021.633610 34040505 PMC8141555

[B31] Juźwik C. A. S Drake S Zhang Y. Paradis-Isler N. Sylvester A. Amar-Zifkin A.. (2019). microRNA dysregulation in neurodegenerative diseases: A systematic review. Prog. Neurobiol. 182, 101664. 10.1016/j.pneurobio.2019.101664 31356849

[B32] Kaltschmidt B. Baeuerle P. A. Kaltschmidt C. (1993). Potential involvement of the transcription factor NF-kappa B in neurological disorders. Mol Aspects Med. 14, 171–190. 10.1016/0098-2997(93)90004-w 8264332

[B33] Kaltschmidt B. Kaltschmidt C. (2009). NF-kappaB in the nervous system. Cold Spring Harb. Perspect. Biol. 1, a001271. 10.1101/cshperspect.a001271 20066105 PMC2773634

[B34] Katti A. Diaz B. J. Caragine C. M. Sanjana N. E. Dow L. E. (2022). CRISPR in cancer biology and therapy. Nat. Rev. Cancer. 22, 259–279. 10.1038/s41568-022-00441-w 35194172

[B35] Kaur U. Banerjee P. Bir A. Sinha M. Biswas A. Chakrabarti S. (2015). Reactive oxygen species, redox signaling and neuroinflammation in Alzheimer's disease: the NF-κB connection. Curr. Top Med. Chem. 15, 446–457. 10.2174/1568026615666150114160543 25620241

[B36] Kittur S. Hoh J. Endo H. Tourtellotte W. Weeks B. S. Markesbery W.. (1994). Cytoskeletal neurofilament gene expression in brain tissue from Alzheimer's disease patients. Decrease in NF-L and NF-M message. J. Geriatr. Psychiatry Neurol. 7, 153–158. 10.1177/089198879400700305 7522458

[B37] Kumar V. (2019). Toll-like receptors in the pathogenesis of neuroinflammation. J. Neuroimmunol. 332, 16–30. 10.1016/j.jneuroim.2019.03.012 30928868

[B38] Lane C. A. Hardy J. Schot J. M. (2018). Alzheimer's disease. Eur. J. Neurol. 25, 59–70. 10.1111/ene.13439 28872215

[B39] Lee Y. Kang H. Lee B. Zhang Y. Kim Y. Kim S.. (2017). Integrative analysis of brain region-specific SHANK3 interactomes for understanding the heterogeneity of neuronal pathophysiology related to SHANK3 mutations. Front. Mol. Neurosci. 10, 110. 10.3389/fnmol.2017.00110 28469556 PMC5395616

[B40] Li C. L. Liu X. H. Qiao Y. Ning L. N. Li W. J. Sun Y. S. (2020a). Allicin alleviates inflammation of diabetic macroangiopathy via the Nrf2 and NF-kB pathway. Eur. J. Pharmacol. 876, 173052. 10.1016/j.ejphar.2020.173052 32135124

[B41] Li L. Xu Y. Zhao M. Gao Z. (2020b). Neuro-protective roles of long non-coding RNA MALAT1 in Alzheimer's disease with the involvement of the microRNA-30b/CNR1 network and the following PI3K/AKT activation. Exp. Mol. Pathol. 117, 104545. 10.1016/j.yexmp.2020.104545 32976819

[B42] Li Y. Y. Cui J. G. Dua P. Pogue A. I. Bhattacharjee S. Lukiw W. J. (2011). Differential expression of miRNA-146a-regulated inflammatory genes in human primary neural, astroglial and microglial cells. Neurosci. Lett. 499, 109–113. 10.1016/j.neulet.2011.05.044 21640790 PMC3713470

[B43] Liu S. Fan M. Zheng Q. Hao S. Yang L. Xia Q.. (2022). microRNAs in Alzheimer's disease: Potential diagnostic markers and therapeutic targets. Biomed. Pharmacother. 148, 112681. 10.1016/j.biopha.2022.112681 35177290

[B44] Lu B. Guo H. Zhao J. Wang C. Wu G. Pang M.. (2010). Increased expression of iASPP, regulated by hepatitis B virus X protein-mediated NF-κB activation, in hepatocellular carcinoma. Gastroenterology. 139, 2183–2194.e5. 10.1053/j.gastro.2010.06.049 20600029

[B45] Lukiw W. J. (2012). NF-κB-regulated micro RNAs (miRNAs) in primary human brain cells. Exp. Neurol. 235, 484–490. 10.1016/j.expneurol.2011.11.022 22138609 PMC3321120

[B46] Lukiw W. J. (2020). microRNA-146a signaling in Alzheimer's disease (AD) and prion disease (PrD). Front. Neurol. 11, 462. 10.3389/fneur.2020.00462 32670176 PMC7331828

[B47] Lukiw W. J. Bazan N. G. (1998). Strong nuclear factor-kappaB-DNA binding parallels cyclooxygenase-2 gene transcription in aging and in sporadic Alzheimer's disease superior temporal lobe neocortex. J. Neurosci. Res. 53, 583–592. 10.1002/(SICI)1097-4547(19980901)53:5<583:AID-JNR8>3.0.CO;2-5. 9726429

[B48] Lukiw W. J. Cui J. G. Marcheselli V. L. Bodker M. Botkjaer A. Gotlinger K.. (2005). A role for docosahexaenoic acid-derived neuroprotectin D1 in neural cell survival and Alzheimer disease. J. Clin. Invest. 115, 2774–2783. 10.1172/JCI25420 16151530 PMC1199531

[B49] Lukiw W. J. Zhao Y. Cui J. G. (2008). An NF-kB-sensitive micro RNA-146a-mediated inflammatory circuit in Alzheimer disease and in stressed human brain cells. J. Biol. Chem. 283, 31315–31322. 10.1074/jbc.M805371200 18801740 PMC2581572

[B50] McLachlan D. R. C. Lukiw W. J. Kruck T. P. (1990). Aluminum, altered transcription, and the pathogenesis of Alzheimer's disease. Environ. Geochem. Health. 12, 103–114. 10.1007/BF01734059 24202576

[B51] Mirza F. J. Zahid S. (2018). The role of synapsins in neurological disorders. Neurosci. Bull. 34, 349–358. 10.1007/s12264-017-0201-7 29282612 PMC5856722

[B52] Miyoshi E. Bilousova T. Melnik M. Fakhrutdinov D. Poon W. W. Vinters H. V.. (2021). Exosomal tau with seeding activity is released from Alzheimer's disease synapses, and seeding potential is associated with amyloid beta. Lab Invest. 101, 1605–1617. 10.1038/s41374-021-00644-z 34462532 PMC8590975

[B53] Natoli R. Fernando N. (2018). microRNA as therapeutics for age-related macular degeneration. Adv. Exp. Med. Biol. 1074, 37–43. 10.1007/978-3-319-75402-4_5 29721925

[B54] Olajide O. A. Sarker S. D. (2020). Alzheimer's disease: natural products as inhibitors of neuroinflammation. Inflammopharmacology. 28, 1439–1455. 10.1007/s10787-020-00751-1 32930914 PMC7572326

[B55] Olufunmilayo E. O. Holsinger R. M. D. (2022). Variant TREM2 signaling in Alzheimer's disease. J. Mol. Biol. 434, 167470. 10.1016/j.jmb.2022.167470 35120968

[B56] Perot B. P. Ménager M. M. (2020). Tetraspanin 7 and its closest paralog tetraspanin 6: membrane organizers with key functions in brain development, viral infection, innate immunity, diabetes and cancer. Med. Microbiol. Immunol. 209, 427–436. 10.1007/s00430-020-00681-3 32468130

[B57] Pires B. R. B. Binato R. Ferreira G. M. Cecchini R. Panis C. Abdelhay E. (2018). NF-kappaB regulates redox status in breast cancer subtypes. Genes (Basel).9, 320. 10.3390/genes9070320 29949949 PMC6070792

[B58] Pogue A. I. Cui J. G. Li Y. Y. Zhao Y. Culicchia F. Lukiw W. J. (2010). Micro RNA-125b (miRNA-125b) function in astrogliosis and glial cell proliferation. Neurosci. Lett. 476, 18–22. 10.1016/j.neulet.2010.03.054 20347935

[B59] Pogue A. I. Lukiw W. J. (2021). microRNA-146a-5p, neurotropic viral infection and prion disease (PrD). Int. J. Mol. Sci. 22, 9198. 10.3390/ijms22179198 34502105 PMC8431499

[B60] Pogue A. I. Zhao Y. Lukiw W. J. (2022). microRNA-146a as a biomarker for transmissible spongiform encephalopathy. Folia Neuropathol. 60, 24–34. 10.5114/fn.2022.113561 35359143

[B61] Rastegar-Moghaddam S. H. Ebrahimzadeh-Bideskan A. Shahba S. Malvandi A. M. Mohammadipour A. (2022). Roles of the miR-155 in neuroinflammation and neurological disorders: a potent biological and therapeutic target. Cell. Mol. Neurobiol. 10.1007/s10571-022-01200-z 35107690 PMC11415209

[B62] Recabarren D. Alarcón M. (2017). Gene networks in neurodegenerative disorders. Life Sci. 183, 83–97. 10.1016/j.lfs.2017.06.009 28623007

[B63] Sen R. Baltimore D. (1986). Multiple nuclear factors interact with the immunoglobulin enhancer sequences. Cell. 46, 705–716. 10.1016/0092-8674(86)90346-6 3091258

[B64] Singh S. Sahu K. Singh C. Singh A. (2022). Lipopolysaccharide induced altered signaling pathways in various neurological disorders. Naunyn Schmiedebergs Arch. Pharmacol. 395, 285–294. 10.1007/s00210-021-02198-9 34989812

[B65] Slota J. A. Booth S. A. (2019). MicroRNAs in neuroinflammation: implications in disease pathogenesis, biomarker discovery and therapeutic applications. Noncoding RNA. 5, 35. 10.3390/ncrna5020035 31022830 PMC6632112

[B66] Song Y. Hu M. Zhang J. Teng Z. Q. Chen C. (2021). A novel mechanism of synaptic and cognitive impairments mediated via microRNA-30b in Alzheimer's disease. EBioMedicine. 39, 409–421. 10.1016/j.ebiom.2018.11.059 30522932 PMC6354659

[B67] Sun G. Y. Simonyi A. Fritsche K. L. Chuang D. Y. Hannink M. Gu Z.. (2018). Docosahexaenoic acid (DHA): an essential nutrient and a nutraceutical for brain health and diseases. Prostaglandins Leukot. Essent. Fatty Acids. 136, 3–13. 10.1016/j.plefa.2017.03.006 28314621 PMC9087135

[B68] Taganov K. D. Boldin M. P. Chang K. J. Baltimore D. (2006). NF-kappaB-dependent induction of microRNA miRNA-146a, an inhibitor targeted to signaling proteins of innate immune responses. Proc. Natl. Acad. Sci. USA. 103, 12481–12486. 10.1073/pnas.0605298103 16885212 PMC1567904

[B69] Tahami Monfared A. A. Byrnes M. J. White L. A. Zhang Q. (2022). Alzheimer's disease: epidemiology and clinical progression. Neurol. Ther. 11, 553–569. 10.1007/s40120-022-00338-8 35286590 PMC9095793

[B70] Trejo-Lopez J. A. Yachnis A. T. Prokop S. (2021). Neuropathology of Alzheimer's disease. Neurotherapeutics. 10.1007/s13311-021-01146-y 34729690 PMC9130398

[B71] Turk A. Kunej T. Peterlin B. (2021). microRNA-target interaction regulatory network in Alzheimer's disease. J. Pers. Med. 11, 1275. 10.3390/jpm11121275 34945753 PMC8708198

[B72] Uddin M. S. Hasana S. Ahmad J. Hossain M. F. Rahman M. M. Behl T.. (2021). Anti-Neuroinflammatory potential of polyphenols by inhibiting NF-κB to halt Alzheimer's disease. Curr. Pharm. Des. 27, 402–414. 10.2174/1381612826666201118092422 33213314

[B73] Valeri A. Chiricosta L. Calcaterra V. Biasin M. Cappelletti G. Carelli S.. (2021). Transcriptomic analysis of HCN-2 cells suggests connection among oxidative stress, senescence, and neuron death after SARS-CoV-2 infection. Cells. 10, 2189. 10.3390/cells10092189 34571838 PMC8472605

[B74] Wang P. Liu Z. Zhang X. Li J. Sun L. Ju Z.. (2018). CRISPR/Cas9-mediated gene knockout reveals a guardian role of NF-κB/RelA in maintaining the homeostasis of human vascular cells. Protein Cell. 9, 945–965. 10.1007/s13238-018-0560-5 29968158 PMC6208479

[B75] Wu Y. Wang M. Yin H. Ming S. Li X. Jiang G.. (2021). TREM-2 is a sensor and activator of T cell response in SARS-CoV-2 infection. Sci Adv. 7, eabi6802. 10.1126/sciadv.abi6802 34878838 PMC8654301

[B76] Xu H. Zhang J. Shi X. Li X. Zheng C. (2021). NF-κB inducible miR-30b-5p aggravates joint pain and loss of articular cartilage via targeting SIRT1-FoxO3a-mediated NLRP3 inflammasome. Aging. 13, 20774–20792. 10.18632/aging.203466 34455406 PMC8436920

[B77] Yoon S. Kim S. E. Ko Y. Jeong G. H. Lee K. H. Lee J.. (2022). Differential expression of microRNAs in Alzheimer's disease: a systematic review and meta-analysis. Mol. Psychiatry. 27, 2405–2413. 10.1038/s41380-022-01476-z 35264731

[B78] Yu J. Yuan X. Chen H. Chaturvedi S. Braunstein E. M. Brodsky R. A. (2020). Direct activation of the alternative complement pathway by SARS-CoV-2 spike proteins is blocked by factor D inhibition. Blood. 136, 2080–2089. 10.1182/blood.2020008248 32877502 PMC7596849

[B79] Zhan X. Hakoupian M. Jin L. W. Sharp F. R. (2021). Lipopolysaccharide, identified using an antibody and by PAS Staining, is associated with corpora amylacea and white matter injury in Alzheimer's disease and aging brain. Front. Aging Neurosci. 13, 705594. 10.3389/fnagi.2021.705594 34899263 PMC8652352

[B80] Zhan X. Stamova B. Sharp F. R. (2018). Lipopolysaccharide associates with amyloid plaques, neurons and oligodendrocytes in Alzheimer's disease brain: a review. Front. Aging Neurosci. 10, 42. 10.3389/fnagi.2018.00042 29520228 PMC5827158

[B81] Zhang Q. Lenardo M. J. Baltimore D. (2017). 30 Years of NF-κB: a blossoming of relevance to human pathobiology. Cell. 168, 37–57. 10.1016/j.cell.2016.12.012 28086098 PMC5268070

[B82] Zhao Y. Arceneaux L. Culicchia F. Lukiw W. J. (2021). Neurofilament Light (NF-L) chain protein from a highly polymerized structural component of the neuronal cytoskeleton to a neurodegenerative disease biomarker in the periphery. HSOA J. Alzheimer's Neurodegener Dis. 7, 056. 10.24966/AND-9608/100056 Epub 2021 Oct 13. 34881359 PMC8651065

[B83] Zhao Y. Bhattacharjee S. Jones B. M. Dua P. Alexandrov P. N. Hill J. M.. (2013). Regulation of TREM2 expression by an NF-κB-sensitive miRNA-34a. Neuroreport 24, 318–323. 10.1097/WNR.0b013e32835fb6b0 23462268 PMC4072209

[B84] Zhao Y. Bhattacharjee S. Jones B. M. Hill J. M. Dua P. Lukiw W. J. (2014). Regulation of neurotropic signaling by the inducible, NF-kB-sensitive miRNA-125b in Alzheimer's disease (AD) and in primary human neuronal-glial (HNG) cells. Mol. Neurobiol. 50, 97–106. 10.1007/s12035-013-8595-3 24293102 PMC4038663

[B85] Zhao Y. Jaber V. R. LeBeauf A. Sharfman N. M. Lukiw W. J. (2019b). microRNA-34a (miRNA-34a) mediated down-regulation of the post-synaptic cytoskeletal element SHANK3 in sporadic Alzheimer's disease (AD). Front. Neurol. 10:28. 10.3389/fneur.2019.00028 30792687 PMC6374620

[B86] Zhao Y. Lukiw W. J. (2013). TREM2 signaling, miRNA-34a and the extinction of phagocytosis. Front. Cell. Neurosci. 7, 131. 10.3389/fncel.2013.00131 24009555 PMC3756624

[B87] Zhao Y. Lukiw W. J. (2018). Bacteroidetes neurotoxins and inflammatory neurodegeneration. Mol. Neurobiol. 55, 9100–9107. 10.1007/s12035-018-1015-y 29637444

[B88] Zhao Y. Sharfman N. M. Jaber V. R. Lukiw W. J. (2019a). Down-regulation of essential synaptic components by GI-tract microbiome-derived lipopolysaccharide (LPS) in LPS-treated human neuronal-glial (HNG) cells in primary culture: relevance to Alzheimer's disease (AD). Front. Cell. Neurosci. 13, 314. 10.3389/fncel.2019.00314 31354434 PMC6635554

[B89] Zingale V. D. Gugliandolo A. Mazzon E. (2021). miRNA-155: an important regulator of neuroinflammation. Int. J. Mol. Sci. 23, 90. 10.3390/ijms23010090 35008513 PMC8745074

